# *Educational Resource Review:* Utilising social science and behaviour change in antimicrobial stewardship programmes: improving healthcare

**DOI:** 10.1093/jacamr/dlaa103

**Published:** 2020-12-21

## Abstract

**Graphical Abstract:**
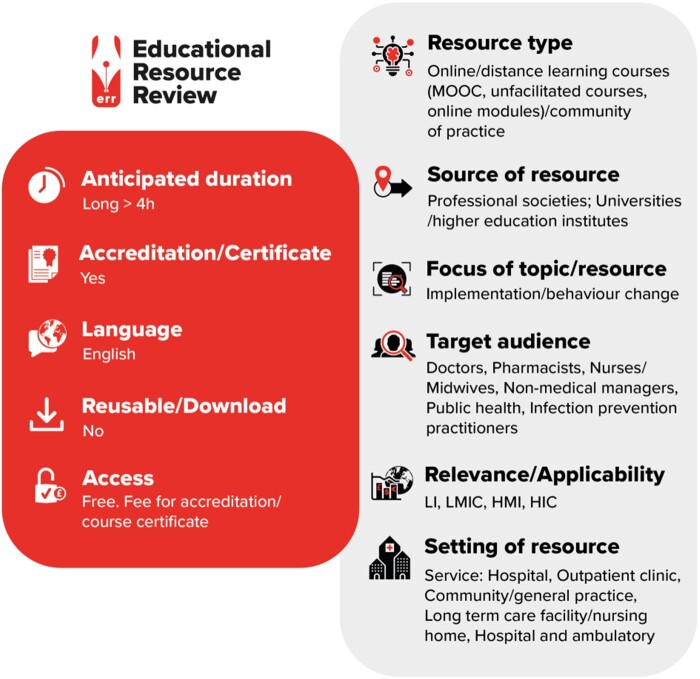

LI, low-income countries; LMIC, low- and middle-income countries; HMI, high- and middle-income countries; HIC, high-income countries.

**Resource web link:**  https://www.futurelearn.com/courses/behaviour-change (Full classification scheme available at: http://bsac.org.uk/wp-content/uploads/2019/03/Educational-resource-review-classification-scheme.pdf)

**WHO region and country (World Bank):** European Region, UK (HIC)

## Peer review commentary

This excellent 2 week course serves as a great introduction to the importance of behaviour change in designing and implementing interventions in antimicrobial stewardship programmes. Beginning with a discussion of why many interventions in this area are not as successful as expected, the concepts of behavioural diagnosis and interventions are framed in the context of a major review of the literature and its findings. Learners may not be familiar with models of behaviour theory such as the capability, opportunity, motivation and behaviour (COM-B) model, but the concepts are explained well through narrated PowerPoint slides and there are plenty of exercises and references for learners to increase their knowledge.

There are links to the related massive open online course (MOOC) on Antimicrobial Stewardship, which many learners might already have undertaken and be familiar with, however, if not then learners would also need to register for that course in order to learn more about the interventions in the South African hospitals. There are some marked differences in the presenting styles, tone and speed of delivery and the sound quality on some of the excerpts could be better.

This is a highly credible resource, produced by a leading professional society in conjunction with experts working in the field. While the narrated PowerPoint slides are very informative and useful, it was engaging to see some role play taking place of a meeting between the medical director and members of the antimicrobial stewardship team; perhaps more could have been made of this and perhaps some of the intervention functions that learners are encouraged to identify and apply to their interventions could have been built into more role-play scenarios.

Learners accessing this resource would also benefit from completing the other MOOC on Antimicrobial Stewardship: Managing Antibiotic Resistance (https://www.futurelearn.com/courses/antimicrobial-stewardship), as the two courses are well aligned.

Overall, this is an excellent and interesting introduction to the importance of behaviour, and ways to change it, in antimicrobial stewardship. It will be useful for healthcare professions of all disciplines and stages of their career.

